# Peroxisomes in Alzheimer’s disease: emerging mechanistic insights and therapeutic opportunities

**DOI:** 10.3389/fnagi.2026.1960320

**Published:** 2026-09-23

**Authors:** Tao Liu, Yiran Liu, Yichi Zhang, Jin Wang, Yulin Ren, Shujun Fu, Li Han, Pengcheng Jiang, Jie Chen, Yuanzhi Li, Huaiqing Luo

**Affiliations:** 1 Department of Pathophysiology, School of Basic Medical Sciences, Hunan Normal University, Changsha, China; 2 Key Laboratory of Model Animals and Stem Cell Biology in Hunan Province, Hunan Normal University, Health Science Center, Changsha, Hunan, China; 3 Department of Physiology, School of Basic Medical Sciences, Hunan Normal University, Changsha, China; 4 Hunan Provincial Key Laboratory of New Pharmaceutical Preparation, Changsha Medical University, Changsha, Hunan, China; 5 Department of Neurosurgery, Affiliated Hengyang Hospital of Hunan Normal University and Hengyang Central Hospital, Hengyang, Hunan, China

**Keywords:** Alzheimer’s disease, lipid metabolism, mitochondria, neuroinflammation, oxidative stress, peroxisome, PPAR

## Abstract

Alzheimer’s disease (AD) is characterized by amyloid-*β* (Aβ) deposition, tau pathology, synaptic injury, neuroinflammation, oxidative stress, and disrupted organellar homeostasis. Peroxisomes maintain lipid and redox balance through very-long-chain fatty acid (VLCFA) *β*-oxidation, plasmalogen biosynthesis, and reactive oxygen species (ROS) metabolism. This Review evaluates how peroxisomal alterations may modulate APP processing and Aβ clearance, tau kinase/phosphatase balance, neuroinflammation, oxidative stress, and peroxisome-mitochondria crosstalk. We distinguish human correlational evidence from intervention studies in AD models and mechanistic extrapolation from primary peroxisomal disorders. Current evidence supports a context-dependent vulnerability or amplifier model rather than a universal causal pathway. Peroxisome-targeted interventions remain investigational, and no validated biomarker currently identifies an AD subgroup for such treatment.

## 1 Introduction

Alzheimer’s disease (AD) ranks among the most prevalent neurodegenerative disorders. Clinically, it presents with progressive memory deterioration, waning executive function, impairments in language and visuospatial orientation, and, eventually, the loss of independent daily living. Its cardinal neuropathological features comprise intracellular neurofibrillary tangles derived from hyperphosphorylated tau, extracellular plaques deposited by amyloid-β (Aβ), alongside synaptic attrition and neuronal loss (De Strooper and Karran, 2016; Long and Holtzman, 2019). As the research paradigm has shifted from models centered on isolated pathological hallmarks toward a framework of multifactorial network dysfunction, metabolic disturbances, organelle crosstalk, immune-mediated inflammation, senescence, and oxidative stress have come to the fore as pivotal determinants of AD heterogeneity and the variable responses to therapeutic interventions (Scheltens et al., 2021).

According to a 2025 report from the World Health Organization, approximately 57 million people worldwide were living with dementia in 2021, with close to 10 million new cases emerging each year. Alzheimer’s disease is estimated to account for 60–70% of all dementia cases, and dementia remains a primary driver of disability and dependency among older adults (World Health Organization, 2025). Projections from the Global Burden of Disease study further indicate that the global prevalence of dementia could escalate from roughly 57.4 million in 2019 to nearly 153 million by 2050, powerfully illustrating the immense strain that healthcare systems, caregiving networks, and social support infrastructures will face over the coming three decades (Nichols et al., 2022).

Recent clinical advances in Aβ-targeting antibodies have introduced disease-modifying treatment for selected patients. Lecanemab received traditional U.S. Food and Drug Administration (FDA) approval in 2023, and donanemab-azbt (Kisunla) was approved on 2 July 2024 for patients with mild cognitive impairment or mild dementia-stage AD with confirmed amyloid pathology (Food and Drug Administration, 2023, 2024; Sims et al., 2023). Their use remains limited by eligibility requirements, modest average clinical effects, amyloid-related imaging abnormalities, and substantial diagnostic and monitoring burdens.

Peroxisomes are dynamic, single-membrane-bound organelles ubiquitously distributed throughout eukaryotic cells, containing abundant oxidases, catalase, and lipid-metabolizing enzymes. Their functions encompass the β-oxidation of very-long-chain fatty acids (VLCFAs), the degradation of branched-chain fatty acids, bile acid synthesis, and the biosynthesis of ether phospholipids—notably plasmalogens. Moreover, via catalase (CAT) and related enzymes, peroxisomes are instrumental in sustaining local reactive oxygen species (ROS) homeostasis (Cipolla and Lodhi, 2017; Fransen et al., 2012; Wanders and Waterham, 2006).

These functions are of particular relevance to the nervous system, where membrane lipid composition, myelin integrity, synaptic plasticity, and redox homeostasis are intimately interconnected. Consequently, peroxisomal dysfunction may simultaneously impair multiple facets of neural cell physiology. Within the central nervous system, peroxisomes establish an integrated metabolic network with the endoplasmic reticulum, mitochondria, lipid droplets, and lysosomes (Islinger et al., 2018; Schrader et al., 2016; Wanders et al., 2016). Thus, peroxisomal abnormalities can concurrently disrupt membrane architecture, energy metabolism, reactive oxygen species (ROS) signaling, and inflammatory responses.

Reduced plasmalogen levels, diminished peroxisomal enzyme activities, enhanced lipid peroxidation, and elevated oxidative stress have been consistently documented in both Alzheimer’s disease (AD) brain tissue and animal models (Chan et al., 2012; Grimm et al., 2011; Han et al., 2001; Igarashi et al., 2011; Kou et al., 2011). Furthermore, peroxisome-regulated nuclear receptors—particularly the peroxisome proliferator-activated receptors (PPARs)—are closely intertwined with lipid metabolism, insulin sensitivity, inflammatory suppression, and mitochondrial function (Heneka et al., 2014), rendering them promising targets for drug repurposing and novel therapeutic discovery.

This focused narrative Review examines peroxisomal lipid metabolism, redox regulation, quality control, neuroimmune signaling, and peroxisome-mitochondria crosstalk in AD. PubMed-indexed human, animal, and cellular studies were considered, with emphasis on work published from 2022 to August 2026 and earlier foundational studies. Evidence is explicitly categorized as human association, intervention in AD models, or mechanistic extrapolation from non-AD systems. Because this is not a systematic review, the synthesis is hypothesis-generating and causal language is reserved for direct experimental manipulation.

## 2 Peroxisomal organization and metabolic roles

### 2.1 Peroxisomal ultrastructure and key enzymes

Peroxisomes are typically spherical or ovoid vesicles, ranging from 0.2 to 1.5 μm in diameter, delimited by a single membrane and harboring a rich array of redox and lipid-metabolic enzymes (Lodhi and Semenkovich, 2014). Their biogenesis proceeds via two principal pathways: division and proliferation of pre-existing peroxisomes, and *de novo* formation. Division, the predominant route, is orchestrated by the Pex11 protein, whereas *de novo* biogenesis entails the generation of pre-peroxisomal vesicles from other organelles—notably mitochondria and the endoplasmic reticulum (ER)—which subsequently mature into fully functional peroxisomes (Mast et al., 2018; Schrader et al., 2012; Sugiura et al., 2017). Unlike mitochondria, peroxisomes lack a genome; all of their proteins are nuclear-encoded, synthesized in the cytosol, and imported via specialized targeting machineries.

A hallmark of peroxisomes is their capacity to both generate and remove ROS. During fatty acid β-oxidation, acyl-CoA oxidase 1 (ACOX1) transfers electrons to oxygen and generates hydrogen peroxide (H₂O₂), whereas catalase (CAT) decomposes H₂O₂ and limits oxidative injury (Antonenkov et al., 2009; Lismont et al., 2019).

ACOX1 and CAT are functionally coupled components of peroxisomal redox homeostasis. Low H₂O₂ concentrations can participate in cellular signaling, whereas excessive or poorly compartmentalized H₂O₂ promotes oxidative damage. These general redox principles are well established, but they should not be attributed to a clinical pioglitazone study in AD (Beach et al., 2012; Bonekamp et al., 2009) (Figure 1).

**Figure 1:**
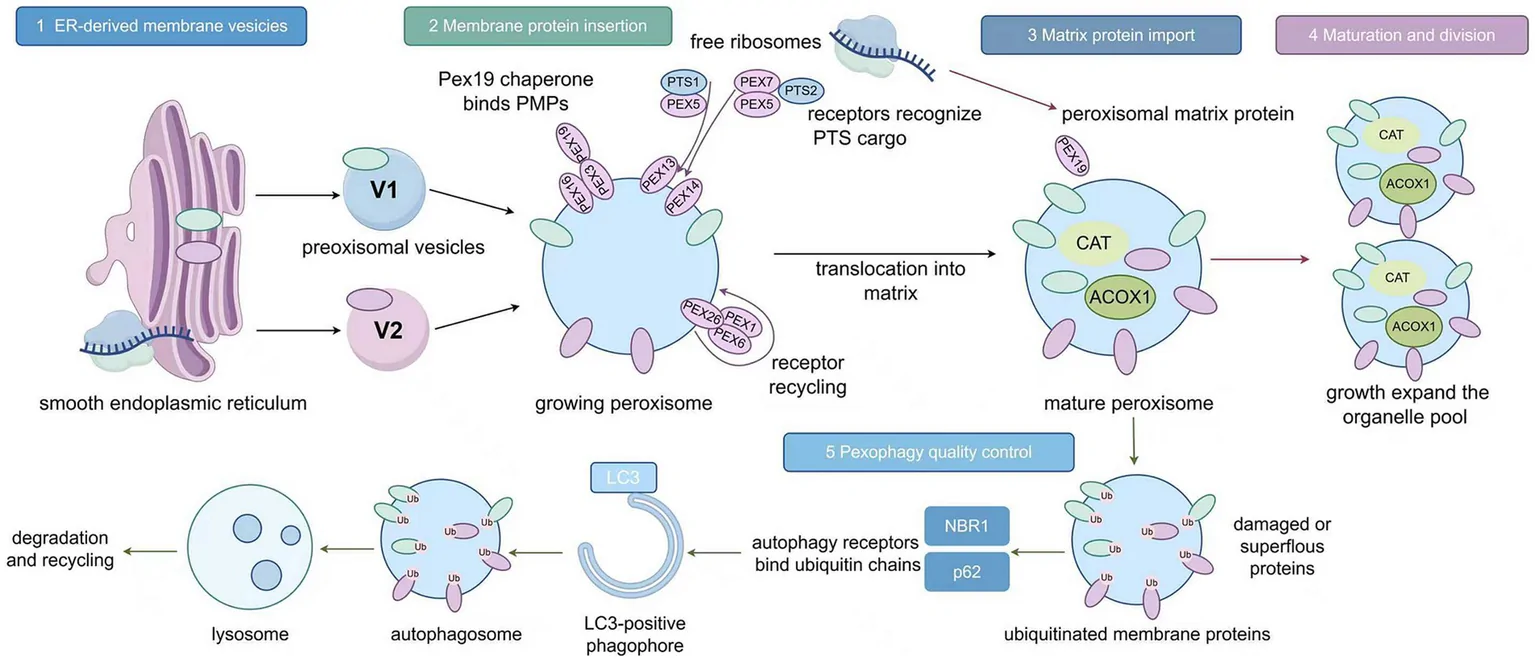
Peroxisome biogenesis and pexophagy. Nuclear-encoded PEX proteins are synthesized on free ribosomes and support membrane assembly, matrix-protein import, growth, and division. Damaged peroxisomes are ubiquitinated and recognized by selective-autophagy receptors before lysosomal degradation. Biogenesis and clearance must remain balanced to maintain a functional peroxisome pool.

### 2.2 Peroxisomal biogenesis and pexophagy: PEX gene regulation and autophagic clearance

Peroxisomal biogenesis is orchestrated by peroxins, the products of PEX genes (Fujiki et al., 2014; Islinger et al., 2018; Lazarow, 2003). Among these, PEX3, PEX16, and PEX19 are primarily responsible for targeting membrane proteins and facilitating membrane formation, whereas PEX5 and PEX7 recognize matrix proteins bearing peroxisomal targeting signals type 1 (PTS1) and type 2 (PTS2), respectively, and cooperate with PEX13 and PEX14 during protein import (Fujiki et al., 2014; Islinger et al., 2018; Lazarow, 2003). Thus, peroxisomes are not static “metabolic vesicles” but rather dynamic organelles that continuously undergo biogenesis, maturation, division, and turnover.

Pexophagy selectively removes damaged or surplus peroxisomes. Ubiquitinated peroxisomal membrane proteins can recruit NBR1 and p62/SQSTM1, which link cargo to LC3-positive autophagosomes for lysosomal degradation (Till et al., 2012). Insufficient pexophagy may allow oxidatively damaged peroxisomes to persist, whereas excessive clearance may deplete the functional organelle pool.

### 2.3 Multifaceted physiological functions of peroxisomes

Peroxisomes are indispensable for the degradation of very-long-chain fatty acids (VLCFAs). Unlike shorter-chain fatty acids, VLCFAs cannot be directly oxidized by mitochondria; they must first be imported into peroxisomes, where they are progressively shortened by a series of enzymes—including ACOX1, multifunctional proteins, and thiolases—to generate shorter-chain fatty acyl-CoA derivatives that subsequently enter mitochondria for further oxidation and energy production (Baes et al., 2000). The clinical significance of this pathway is underscored by defects in transporters such as ABCD1, which lead to VLCFA accumulation and neurological impairment, highlighting the critical importance of peroxisomal lipid metabolism for myelin integrity, axonal health, and neuronal function (Berger et al., 2016).

Beyond fatty acid oxidation, peroxisomes participate in the side-chain shortening step of bile acid synthesis and are intimately linked to cholesterol metabolism (Wanders and Waterham, 2006). Of particular relevance to Alzheimer’s disease is their role in plasmalogen synthesis. Plasmalogens are essential constituents of neural membranes and myelin, contributing to antioxidant defense, membrane fluidity regulation, and synaptic vesicle function. Notably, reduced plasmalogen levels have been consistently observed in AD brain tissues and animal models, suggesting that impaired peroxisomal ether lipid biosynthesis may contribute to synaptic degeneration and neural network deterioration (Brites et al., 2004; Goodenowe et al., 2007; Han et al., 2001; Igarashi et al., 2011).

Peroxisomes serve as both sources and sinks of reactive oxygen species (ROS). Under physiological conditions, hydrogen peroxide (H₂O₂) generated by ACOX1 during β-oxidation is efficiently scavenged by catalase (CAT) and related enzymes. However, under conditions of aging, inflammation, or metabolic stress, diminished CAT activity or reduced peroxisomal abundance allows H₂O₂ to diffuse into the cytoplasm, mitochondria, and nucleus, thereby promoting lipid peroxidation, protein carbonylation, and DNA damage (Butterfield and Halliwell, 2019; Lismont et al., 2019). This mechanism is consistent with the early elevation of oxidative damage markers observed in AD brains.

Peroxisomal function depends on coordinated protein import, division, metabolic enzyme activity, and selective turnover. Peroxisome abundance and morphology respond to lipid load and oxidative stress (Fujiki et al., 2014; Smith and Aitchison, 2013). CAT and ACOX1 localization influence H₂O₂ handling, while plasmalogens support neural membrane and myelin stability (Antonenkov et al., 2009; Braverman and Moser, 2012; Dorninger et al., 2017). Pexophagy provides an additional quality-control layer (Till et al., 2012).

## 3 The role of Peroxisomal dysfunction in Alzheimer’s disease

### 3.1 Effects on APP processing and aβ clearance

Aβ is generated by sequential cleavage of amyloid precursor protein (APP) by β and γ-secretases. β-site APP-cleaving enzyme 1 (BACE1) is the principal neuronal β-secretase that initiates amyloidogenic APP processing (Cai et al., 2001; Vassar et al., 1999). Peroxisomes are unlikely to dictate APP cleavage directly; instead, their lipid and redox functions may alter the balance between Aβ production and clearance.

Plasmalogen and cholesterol composition influence lipid-raft organization, APP localization, and secretase access. Oxidative stress can increase BACE1 expression, whereas glial uptake and degradation of Aβ depend on cellular energy and lipid homeostasis. These observations support a modulatory pathway involving membrane composition, redox state, and clearance capacity, but direct evidence that a primary peroxisomal defect initiates amyloid deposition in sporadic human AD remains limited (Di Paolo and Kim, 2011; Ehehalt et al., 2003; Fabelo et al., 2014; Mawuenyega et al., 2010; Simons et al., 1998; Tanzi et al., 2004).

Brain metabolic dysfunction can precede detectable proteinopathy in some cohorts, yet temporal ordering varies across populations and biomarkers. A reciprocal model is therefore more appropriate: metabolic vulnerability may potentiate Aβ pathology, while Aβ can further disrupt lipid and organelle homeostasis (Christodoulou et al., 2026).

### 3.2 Regulation of tau hyperphosphorylation via kinase/phosphatase systems

Tau phosphorylation is controlled by multiple kinases and phosphatases. ROS and lipid-peroxidation products can activate stress-responsive kinases, including GSK-3β and CDK5, and may suppress protein phosphatase 2A. However, most studies have not directly manipulated peroxisomal function while measuring site-specific tau phosphorylation; this pathway should therefore be treated as a mechanistic hypothesis rather than an established causal chain (Hanger et al., 2009; Patrick et al., 1999; van der Kant et al., 2020; Wang and Mandelkow, 2016).

Long-chain fatty-acid imbalance may connect mitochondrial dysfunction, lipid peroxidation, ferroptotic susceptibility, and neuroinflammation. Importantly, saturated and unsaturated species can have divergent effects, so lipid class, compartment, and disease stage must be considered (Alam et al., 2026).

Future studies should co-measure PEX expression, CAT and ACOX1 activity, VLCFAs, plasmalogen species, lipid-peroxidation products, mitochondrial respiration, and site-specific tau phosphorylation after cell-type-specific peroxisomal perturbation.

### 3.3 Cross-species evidence: peroxisomal abnormalities from animal models to AD patient brains

Cross-species evidence supports an association between peroxisomal dyshomeostasis and AD-related pathology, but the strength and direction of the relationship vary by species, model, brain region, and disease stage.

Human evidence is cross-sectional and heterogeneous. Kou et al. (2011) identified peroxisomal and lipid abnormalities in AD brain tissue. A later morphometric pilot study reported region and stage-dependent trajectories of PEX14-positive peroxisomes: density declined in some areas, increased and then declined in others, and remained unchanged elsewhere (Semikasev et al., 2023). These findings argue against a uniform progressive peroxisomal deficit and cannot establish temporal causality.

Experimental systems demonstrate biological plausibility rather than direct equivalence to sporadic AD. Severe peroxisomal disorders, including Zellweger spectrum disorders and X-linked adrenoleukodystrophy (X-ALD), show that impaired peroxisomal biogenesis or β-oxidation can cause white-matter injury and neuroinflammation, but these diseases are not AD phenocopies. In engineered microglial models, peroxisomal β-oxidation defects and VLCFA accumulation induce lipid, lysosomal, autophagic, and disease-associated microglial-like programs (Raas et al., 2023; Tawbeh et al., 2025).

Common-variant AD genome-wide association studies strongly implicate lipid handling and microglial pathways, including APOE, ABCA7, TREM2, and PLCG2, but do not currently establish PEX genes, ACOX1, or ABCD1 as major AD susceptibility loci. This absence does not exclude downstream or rare-variant effects, but it argues against inherited peroxisomal failure as a common primary cause of sporadic AD. Whether observed abnormalities are triggers, amplifiers, compensatory responses, or late-stage accompaniments requires longitudinal human studies and conditional genetic models (Bellenguez et al., 2022) (Table 1).

**Table 1:** Peroxisomal abnormalities and their roles in AD pathophysiology.

Pathological dimension	Peroxisome-related changes	Possible consequences	References
Aβ metabolism	Decreased plasmalogen levels, altered membrane lipid raft structure, and increased ROS	Promotion of amyloidogenic APP processing and impairment of the clearance capacity of glial cells	Di Paolo and Kim, 2011; Grimm et al., 2011
Tau pathology	Activation of kinases such as GSK-3β and CDK5 by ROS and inflammatory signalling	Tau hyperphosphorylation and decreased microtubule stability	Ballatore et al., 2007; Hanger et al., 2009
Synapses and myelin	VLCFA accumulation and insufficient ether lipid synthesis	Decreased membrane fluidity and impaired axonal transport and myelin maintenance	Braverman and Moser, 2012; Wanders et al., 2010
Organelle interactions	Decreased peroxisome-mitochondria metabolic cooperation	Energy stress, altered calcium homeostasis, and potentially increased susceptibility to injury	Schrader et al., 2015; Schrader et al., 2016

## 4 Peroxisomes as modulators of neuroinflammation: implications for AD

### 4.1 Regulation of key inflammatory pathways: NF-κB and the NLRP3 inflammasome

Neuroinflammation contributes to AD progression, but evidence should be separated by model. Aβ, tau fragments, damage-associated molecular patterns, and oxidized lipids can activate microglia and astrocytes. General cell-biological studies show that ROS and lipid stress can regulate NF-κB signaling; direct evidence that peroxisomal dysfunction initiates this pathway in human AD remains limited (Block et al., 2007; Heneka et al., 2015).

In APP/PS1 mice, NLRP3 activation contributes to amyloid-associated pathology (Heneka et al., 2013), but that study did not identify peroxisomes as the initiating lesion. Peroxisomal redox or lipid stress may lower the inflammatory threshold, yet a peroxisome-to-NLRP3 causal chain has not been demonstrated in human AD. This distinction separates AD-specific evidence from mechanisms extrapolated from general inflammasome biology.

### 4.2 Effects on microglial polarization and the release of inflammatory factors

Microglia occupy heterogeneous states that are not adequately represented by a simple M1/M2 dichotomy. Disease-associated microglia (DAM) exhibit transcriptional programs related to phagocytosis, lysosomal function, and lipid processing. DAM is a context-dependent state rather than a synonym for neurotoxicity, and its effects vary with disease stage (Colonna and Butovsky, 2017; Hansen et al., 2018; Keren-Shaul et al., 2017; Li and Barres, 2018; Sarlus and Heneka, 2017; Shi and Holtzman, 2018; Ulland et al., 2017).

Peroxisomal β-oxidation-deficient microglial models develop DAM-like signatures and altered cholesterol, lysosomal, and autophagic pathways (Raas et al., 2023). These findings support a link between peroxisomal metabolism and microglial state, but they arise from engineered non-AD systems and should not be presented as direct evidence of the TREM2-APOE pathway in human AD.

Beyond microglia, astrocytes, the blood–brain barrier (BBB), and the neurovascular unit collectively orchestrate the neuroinflammatory milieu. Reactive astrocytes can be induced by activated microglia, while BBB disruption further aggravates neuroinflammation and heightens neuronal vulnerability (Liddelow et al., 2017; Sweeney et al., 2018; Zlokovic, 2011). Thus, peroxisomal dysfunction may amplify neuroinflammation not only through intrinsic microglial alterations but also by perturbing the broader glial–vascular network.

## 5 The role of peroxisomes in oxidative stress and neurodegeneration

### 5.1 Imbalance in ROS removal mechanisms and oxidative neuronal damage

Oxidative stress is detectable early in AD, but it arises from multiple sources, including mitochondria, inflammatory cells, metals, and protein aggregates. Peroxisomes both generate and remove H₂O₂; reduced CAT capacity, altered ACOX1 flux, or defective protein import may increase oxidative burden. Attribution to peroxisomes therefore requires organelle-resolved measurements rather than general oxidative-stress markers. Oxidative damage has been reported at preclinical and mild cognitive impairment stages, although these markers are not peroxisome-specific (Butterfield and Halliwell, 2019; Nunomura et al., 2001; Praticò et al., 2002; Smith et al., 2000).

Lipid-peroxidation products can modify synaptic, mitochondrial, and cytoskeletal proteins. Plasmalogens may buffer membrane oxidation, so reduced levels could increase vulnerability. However, the sequence from peroxisomal dysfunction to neuronal death is not deterministic. Long-chain fatty-acid composition, mitochondrial function, iron handling, and antioxidant capacity jointly influence lipid-peroxidation and ferroptotic susceptibility (Alam et al., 2026).

## 6 Functional interactions between peroxisomes and mitochondria

### 6.1 Metabolic cooperation and signaling interactions

Peroxisomes shorten VLCFAs and generate metabolites that can be further oxidized by mitochondria. The two organelles also participate in redox signaling, share elements of fission machinery, and communicate through membrane-contact networks (Schrader et al., 2015, 2016).

Mitochondrial respiratory and quality-control defects are well documented in AD, whereas the incremental contribution of peroxisomal impairment is less certain. A combined-stress model predicts reduced ATP availability, increased lipid and ROS burden, and impaired organelle turnover, but direct causal evidence in sporadic human AD is not yet available (Fang et al., 2019; Kerr et al., 2017; Lin and Beal, 2006; Swerdlow, 2018; Wang et al., 2009).

Accordingly, assessment should integrate mitochondrial respiration, lipid profiles, oxidative-stress markers, and organelle quality control while accounting for age, vascular and metabolic risk, APOE genotype, brain region, and cell type.

## 7 Potential of peroxisomes as therapeutic targets for AD

### 7.1 Pharmacological intervention strategies: PPAR agonists and peroxisomal function modulation

The PPAR family links lipid metabolism and inflammatory regulation. PPAR agonists improve metabolic and inflammatory readouts in several AD models, but these effects do not establish clinical efficacy. Fenofibrate is a PPARα agonist with context-dependent preclinical relevance: some experimental work supports PPARα-linked neuroprotective mechanisms, whereas fenofibrate has also increased Aβ42 production in a cell-based screening study (Desvergne and Wahli, 1999; Inestrosa et al., 2013; Kukar et al., 2005; Wójtowicz et al., 2020). No randomized AD trial has established a cognitive benefit of fenofibrate.

Clinical translation has been disappointing. Phase III rosiglitazone trials did not demonstrate cognitive benefit, and the TOMMORROW trial reported that low-dose pioglitazone did not delay mild cognitive impairment due to AD and was terminated for futility (Gold et al., 2010; Burns et al., 2021). No validated biomarker currently identifies a “metabolic-inflammatory AD endophenotype” for PPAR treatment selection; biomarker-guided stratification remains a research proposal rather than an actionable clinical strategy.

### 7.2 Emerging therapeutic strategies: genetic, nutritional, and combination approaches

Genetic and organellar quality-control strategies represent a promising future direction. Theoretically, enhancing PEX gene expression, improving peroxisomal protein import, promoting moderate pexophagy, or augmenting catalase (CAT) activity could all improve peroxisomal homeostasis. However, these approaches face formidable challenges, including blood–brain barrier delivery, cell-type specificity, safety concerns, and the long-term stability of regulatory interventions (Smith and Aitchison, 2013). In the nervous system in particular, excessive enhancement of either peroxisomal proliferation or autophagy may provoke metabolic side effects; hence, precise and titrated regulation is essential.

Plasmalogen supplementation has been examined in small clinical studies with heterogeneous formulations, populations, and outcomes; current evidence is insufficient to establish disease modification. Future lipid-directed trials should document baseline deficiency, central nervous system exposure, and target engagement rather than assume that peripheral supplementation restores neuronal peroxisomal function. A 24-week multicenter randomized trial reported subgroup-dependent cognitive signals after scallop-derived plasmalogen supplementation, but the findings require independent replication and do not establish disease modification (Fujino et al., 2017).

Given the multifactorial nature of AD, combination therapy may offer greater therapeutic promise than monotherapy. Peroxisome-targeting strategies could be rationally integrated with anti-Aβ antibodies, anti-Tau therapies, anti-inflammatory agents, vascular risk factor management, and lifestyle modifications. For instance, while anti-Aβ antibodies reduce plaque burden, concurrent enhancement of lipid metabolism and amelioration of the inflammatory microenvironment via the PPAR/peroxisome axis could, in theory, facilitate neuronal resilience and mitigate the counteracting effects of chronic inflammation on therapeutic efficacy.

## 8 Current challenges and future perspectives

### 8.1 Challenges facing peroxisome research in AD

Despite substantial progress, several critical challenges impede the translation of peroxisomal findings into clinical practice.

First, the causal relationship between peroxisomal dysfunction and AD pathology remains unresolved. Peroxisomal abnormalities may represent early drivers of the disease, or they may emerge as secondary consequences of Aβ/Tau pathology, neuroinflammation, and aging. Cross-sectional analyses of human brain specimens cannot reliably establish temporal precedence, while conventional animal models often fail to fully recapitulate the complexity of sporadic AD.

Second, cell-type specificity is insufficiently characterized. Peroxisomal functions are not uniformly distributed across neurons, oligodendrocytes, astrocytes, and microglia; analyses of bulk brain tissue may therefore obscure critical changes occurring in key cell populations (Kassmann et al., 2007).

Third, targeted therapeutic delivery faces substantial hurdles. Peroxisome-related interventions must cross the blood–brain barrier and achieve effective concentrations within specific cell types, while avoiding off-target effects arising from peripheral lipid metabolism disturbances and excessive hepatic peroxisomal proliferation.

Fourth, clinical translation lacks reliable pharmacodynamic biomarkers. Conventional cognitive scales exhibit slow response times and are influenced by numerous confounding factors, rendering them insufficiently sensitive to detect early improvements in peroxisomal function. While lipidomic profiles, plasmalogen levels, VLCFAs, exosomal peroxisomal proteins, and imaging-based inflammatory indicators hold promise as candidate biomarkers, they have yet to undergo rigorous standardization and validation. Existing human brain and lipidomic studies support candidate-marker development but not current clinical qualification (Kou et al., 2011; Mapstone et al., 2014; Wood et al., 2015).

### 8.2 Future research directions

Future investigations should advance along four interconnected axes.

First, high-resolution spatial profiling. Single-cell sequencing, spatial transcriptomics, and spatial lipidomics should be employed to map peroxisome-related gene expression and lipid metabolism across different disease stages, brain regions, and cell types in AD.

Second, human iPSC-based modeling. Induced pluripotent stem cell (iPSC)-derived neuronal, glial, and brain organoid models should be developed and engineered to incorporate APOEε4 genotype, glucose/lipid metabolic abnormalities, and aging-related stressors, thereby more faithfully recapitulating the sporadic AD environment.

Third, multi-omics biomarker discovery. Lipidomics, proteomics, metabolomics, and inflammomics should be integrated to identify peroxisome-related biomarkers suitable for patient stratification and pharmacodynamic monitoring.

Fourth, precision-stratified clinical trials should be considered only after candidate lipid, inflammatory, or peroxisomal biomarkers have undergone analytical and clinical validation. At present, no biomarker-defined subgroup is established for PPAR or peroxisome-targeted treatment.

Peroxisome research should be integrated with Aβ and tau biology without positioning peroxisomes as a proven central driver. A cross-scale framework can test how membrane lipids influence APP processing, how redox and inflammatory signals interact with tau, and how organelle crosstalk affects synaptic energy balance. In this framework, peroxisomes are candidate context-dependent modifiers within a broader AD network.

## 9 Conclusion

Peroxisomes maintain neural lipid, redox, and organelle homeostasis. Human studies associate peroxisomal and lipid alterations with AD, while experimental systems support plausible links to Aβ homeostasis, tau-regulatory pathways, neuroinflammation, oxidative stress, and mitochondrial function. Current evidence does not establish peroxisomal dysfunction as a necessary, sufficient, or universal cause of AD; it is more defensibly viewed as a context-dependent vulnerability, amplifier, or response within reciprocal metabolic-proteinopathy networks (Figure 2).

**Figure 2:**
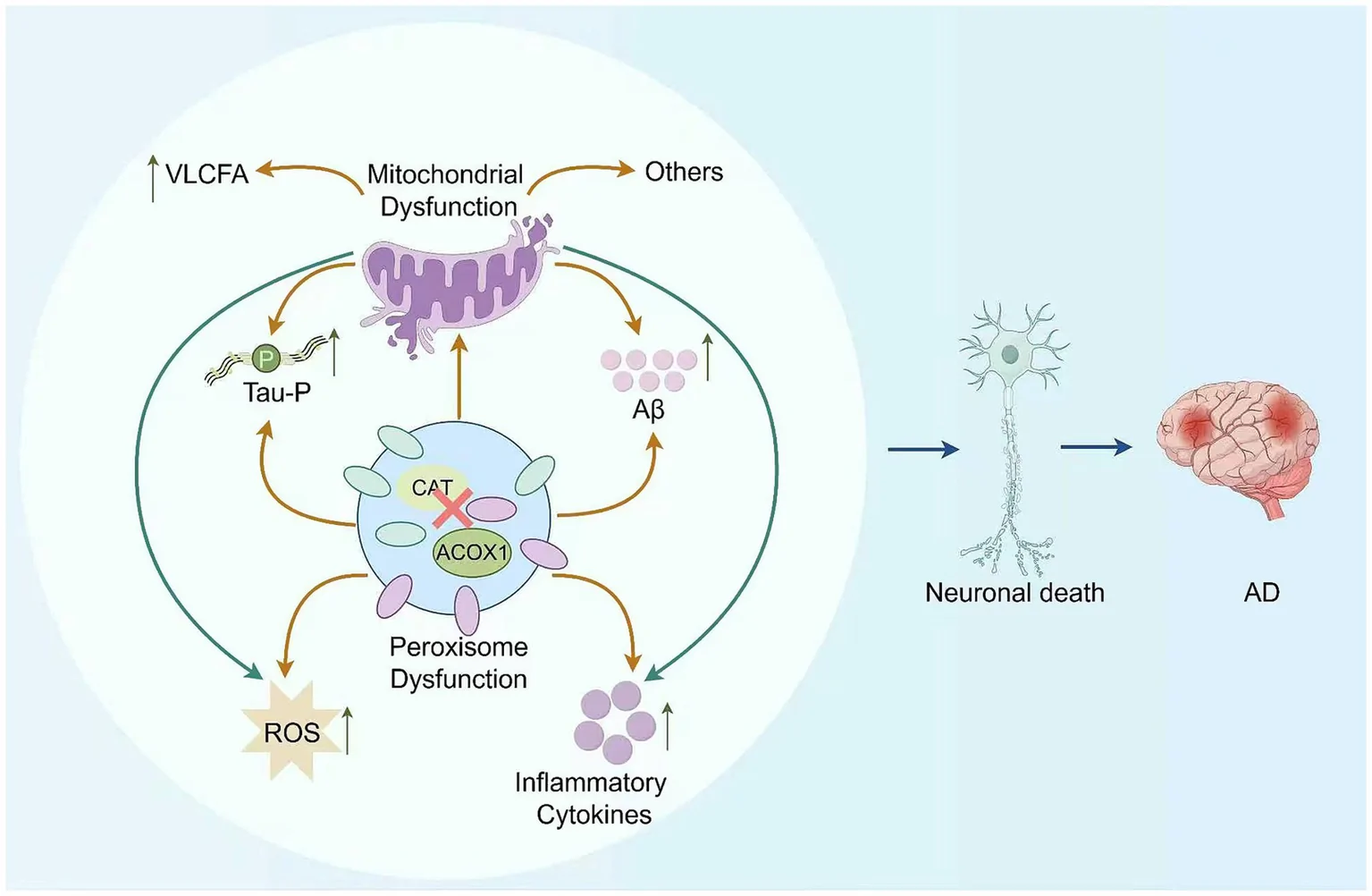
The scheme summarizes the pathways through which peroxisomal dysfunction is proposed to interact with AD-associated processes, including Aβ homeostasis, tau phosphorylation, neuroinflammation, oxidative stress, mitochondrial function, and very-long-chain fatty acid (VLCFA) metabolism. Consistent with the human and experimental evidence discussed in the text, these connections are depicted as reciprocal and context-dependent: peroxisomal dysfunction may act as a vulnerability, amplifier, or response within these networks, and the progression toward neuronal death and AD represents one possible outcome rather than a necessary or universal causal route.

PPAR agonism, lipid modulation, pexophagy regulation, and restoration of peroxisome-mitochondria communication remain investigational. Translation requires longitudinal human evidence, validated target-engagement biomarkers, and adequately powered trials in biologically defined populations.

Peroxisomal biology therefore provides a testable framework for studying lipid-redox-organelle interactions in AD, while substantial uncertainty remains regarding temporal precedence, causal magnitude, and therapeutic tractability.
